# Intravenous-oral itraconazole versus oral posaconazole in preventing invasive fungal diseases for acute leukemia patients

**DOI:** 10.1097/BS9.0000000000000155

**Published:** 2023-03-16

**Authors:** Li Liu, Xiaolei Pei, Runzhi Ma, Yi He, Rongli Zhang, Jialin Wei, Qiaoling Ma, Weihua Zhai, Aiming Pang, Erlie Jiang, Mingzhe Han, Donglin Yang, Sizhou Feng

**Affiliations:** a Hematopoietic Stem Cell Transplantation Center, National Clinical Research Center for Blood Diseases, State Key Laboratory of Experimental Hematology, Haihe Laboratory of Cell Ecosystem, Institute of Hematology & Blood Diseases Hospital, Chinese Academy of Medical Sciences & Peking Union Medical College, Tianjin 300020, China

**Keywords:** Acute leukemia, Antifungal prophylaxis, Invasive fungal disease, Itraconazole, Posaconazole

## Abstract

Invasive fungal diseases (IFDs) are major and lethal infectious complications for patients with neutropenia after chemotherapy. Prophylaxis with intravenous and oral suspended itraconazole (200 mg Q12h intravenously × 2 days followed by 5 mg/kg·d orally in twice) or oral suspension of posaconazole (200 mg Q8h) was administered for preventing IFDs. The only 2 episodes of proven IFDs were not included after propensity-score matching (PSM), while the incidence of possible IFDs was 8.2% (9/110) in itraconazole group and 1.8% (2/110) in posaconazole group, respectively (*P* = .030). In clinical failure analysis, the failure rate of posaconazole group was lower as compared to the itraconazole group (2.7% vs 10.9%, *P* = .016). Both intravenous-oral itraconazole and posaconazole suspension are effective in preventing IFDs, while posaconazole suspension seems more tolerable.

## 1. INTRODUCTION

Invasive fungal diseases (IFDs) are life-threatening infectious complications for patients with acute leukemia (AL) undergoing prolonged and severe neutropenia after chemotherapy.^1–4^ In these patients, IFDs are important causes of morbidity and mortality.^1,5,6^ Although the diagnostic methodology advancing, such as serum β-D-glucan/galactomannan test and high-resolution computer tomography,^3^ IFDs remains not easy to be diagnosed due to their nonspecific clinical manifestations on the early stage. It is important to select appropriate antifungal prophylaxis for immunocompromised patients at high risk of IFDs.^7^ Therefore, antifungal prophylaxis using efficacious and well-tolerated agents is of particular clinical significance.^7,8^ Currently, primary antifungal choices include posaconazole, voriconazole, itraconazole, liposomal amphotericin B, micafungin, and caspofungin.^9,10^ We previously reported that oral itraconazole was inferior to sequential administration of intravenous and oral itraconazole,^11^ which is also recommended by the Chinese guidelines for the diagnosis and treatment of IFD in patients with hematological disorders and cancers (the fifth revision).^10^ As reported, oral suspension of posaconazole is superior to the same formulation of fluconazole or itraconazole in preventing IFDs for patients with hematological malignancies.^12^ However, we rarely know about if the prophylactic efficiency of itraconazole in an intravenous-oral regimen could rival oral posaconazole. To contribute to this issue, we compared itraconazole in an intravenous-oral regimen and oral suspension of posaconazole for primary antifungal prophylaxis (PAP) in terms of efficacy and clinical failures among neutropenic patients with AL after chemotherapy.

## 2. MATERIALS AND METHODS

### 2.1. Patients and study design

In this observational, single-center, retrospective study, 2 cohorts of patients (age ≥13 years old) was compared. Patients with AL were collected from January 1, 2016 to August 31, 2018, if they accepted itraconazole in the intravenous-oral regimen or oral suspension of posaconazole to prevent IFDs after chemotherapy. Patients would be discharged and not analyzed subsequently, thereafter they had documented IFDs and needed long-term antifungal therapy or secondary prophylaxis.

Antifungal prophylaxis consisted of administering either intravenous and oral suspension of itraconazole (200 mg Q12h intravenously × 2 days followed by 5 mg/kg·d orally in twice) or posaconazole suspension (200 mg Q8h). Generally, prophylaxis was started at the same time of chemotherapy and was continued until recovery from neutropenia, occurrence of IFDs, or death of any reason, whichever came first. Besides, Vinca alkaloids was adjusted into half dose for patients with acute lymphocytic leukemia (ALL) in order to avoid drug–drug interactions.

The primary outcome was the rates of IFDs. Secondary outcomes were the rates of clinical failure and adverse events.

### 2.2. Ethics statement

This retrospective study was approved by the Ethics Committee of Blood Diseases Hospital, Chinese Academy of Medical Sciences according to the Declaration of Helsinki (Approval No. NI2020006-EC-1). And the consent has been waived, because we would not reveal patient’s private information or do any harm to them.

### 2.3. Definitions

All clinical records were reviewed carefully for any evidence of IFDs from the beginning to the end of the prophylaxis. Diagnosis and classification of IFD was performed according to the European Organization for Research and Treatment of Cancer and Mycoses Study Group (EORTC/MSG) revised definitions of 2019.^13^ Clinical failure includes: the occurrence of proven or probable IFDs; the receipt of any other systemic antifungal agent for 4 days or more or 10 days in total for suspected IFDs; the occurrence of adverse events possibly or probably related to prophylactic drugs that results in the discontinuation.^12^ All records about adverse events were assessed according to the National Cancer Institute Common Terminology Criteria for Adverse Events Version 5.0. Survival was evaluated for each chemotherapy episode, and the overall survival was analyzed.

### 2.4. Statistical methods

Propensity-score matching (PSM) was applied to identify a cohort of patients with similar baseline characteristics, given the differences between eligible ones in the two groups (Table 1). The propensity score was estimated by a non-parsimonious multivariable logistic-regression model, with the prophylactic drugs as the dependent variable while all variables in Table 1 as covariates. Matching was performed using a 1:1 matching protocol without replacement, with a caliper width equal to 0.05 of the standard deviation of the logit of the propensity score. To assess pre-match imbalance and post-match balance, standardized differences were estimated for all baseline covariates before and after matching. This was carried out using R (version 3.5.3).

**Table 1.** Demographic and clinical characteristics of patients before and after PSM.

Characteristic	Before matching			After matching		
	Posaconazole (N = 130)	Itraconazole (N = 212)	*P* value	Posaconazole (N = 110)	Itraconazole (N = 110)	*P* value
Age (y), median (range)	33.87 (14–66)	35.56 (13–59)	.207	34.54 (14–66)	35.17 (13–59)	.630
Male, no. (%)	61 (46.9)	120 (56.6)	.082	56 (50.9)	57 (51.8)	.893
Primary diagnosis, no. (%)			.040			.487
AML	76 (58.5)	147 (69.3)		71 (64.5)	66 (60.0)	
ALL	54 (41.5)	65 (30.7)		39 (35.5)	44 (40.0)	
Chemotherapy phases			.008			.914
Induction	20 (15.4)	62 (29.2)		20 (18.2)	22 (20.0)	
Re-induction	3 (2.3)	7 (3.3)		3(2.7)	2 (1.8)	
Consolidation	107 (82.3)	143 (67.5)		87 (79.1)	86 (78.2)	
Duration of neutropenia (ANC <0.5 × 10^9^/L) (d), median (range)	14.29 (2.0–82.0)	15.11 (0.0–68.0)	.204	14.755 (2.0–82.0)	14.671 (3.0–68.0)	.776
Duration of severe neutropenia (ANC <0.1 × 10^9^/L) (d), median (range)	8.89 (0.0–54.0)	9.09 (0.0–53.0)	.744	9.391 (0.0–54.0)	9.026 (0.0–53.0)	.432

ALL = acute lymphocytic leukemia, ANC = absolute neutrophil count, AML = acute myeloid leukemia, PSM = propensity-score matching.

In the matched cohort, the chi-square or Fisher exact test and Mann–Whitney *U* test were used to analyze the differences for categorical variables and continuous variables between the two groups. Differences were considered statistically significant if the type I error alpha was <5% (*P* < .05). All *P* values reported are two-sided. Statistical analyses were performed using SPSS software (version 25.0).

## 3. RESULTS

### 3.1. Baseline characteristics

This study identified 342 eligible episodes from 186 patients with AL who met the inclusion criteria, of whom 130 (38.0%) were on posaconazole and 212 (62.0%) on itraconazole in an intravenous-oral regimen. Before PSM, there were differences in several of the baseline variables between the two cohorts. After PSM, 110 episodes on posaconazole and 110 episodes on itraconazole in an intravenous-oral regimen were matched. After matching, the standardized differences were less than 10.0% for all baseline variables between the two groups (Table 1).

### 3.2. Primary outcomes

In the pre-matched cohorts, 2 patients developed proven IFDs (2 candidemia, 0.9%) in the itraconazole group, while none in the posaconazole group. The numbers of probable and possible cases of IFDs were 0 and 3 (2.3%) in the posaconazole group, and 0 and 16 (7.5%) in the itraconazole group (Table S1, http://links.lww.com/BS/A56).

**Figure 1.**
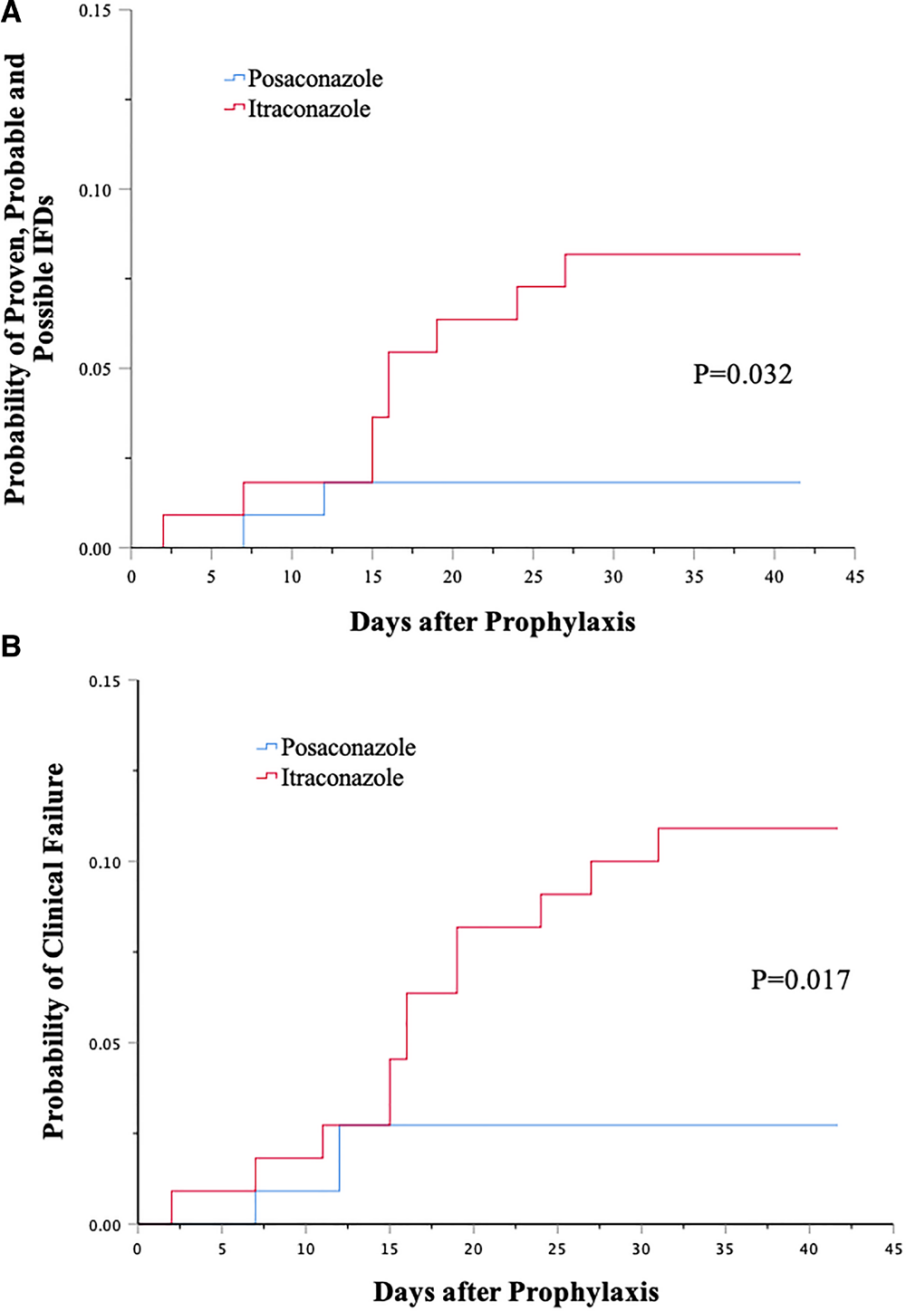
Kaplan–Meier curves for time to proven, probable and possible IFDs (A) and clinical failure (B) over the 42-d period after prophylaxis. *P* values were estimated with the log-rank test. IFD = invasive fungal diseases.

After matching, there were no proven or probable IFDs either in the itraconazole or the posaconazole group. The rates of possible IFDs were 8.2% (itraconazole, 9/110) versus 1.8% (posaconazole, 2/110) in matched cohorts (*P* = .030). As to the patients with proven, probable, and possible IFDs, both in posaconazole cohort were undergoing consolidation chemotherapy, and 4 of 9 in itraconazole cohort were in induction or re-induction chemotherapy. Kaplan–Meier analysis of the time to proven, probable, and possible IFDs during the 42-day period showed a significant difference in favor of posaconazole (*P* = .032) (**Fig. 1A**).

### 3.3. Secondary outcomes

The rate of clinical failure was lower in posaconazole group of the matched cohorts (2.7% vs 10.9%, *P* = .016). Of 110 episodes in the posaconazole group, 3 (2.7%) received empirical and systemic antifungal treatment, as did 11 of 110 patients (10%) in the itraconazole group (*P* = .027). Besides, the prophylaxis using itraconazole of 1 episode was discontinued due to incomplete intestinal obstruction, which may relate to the potentially hazardous neurotoxic interaction of azoles with Vinca alkaloids.^14^ During the 42-day period, the analysis of the time to clinical failure revealed a significant difference in favor of posaconazole over itraconazole (*P* = .017; **Fig. 1B**).

Adverse events were compared between matched groups. One episode (0.9%) with rashes, 1 (0.9%) with diarrhea, 1 (0.9%) with rashes and diarrhea, and 2 (1.8%) with liver dysfunction were noted in the posaconazole group. In the itraconazole group, rashes were reported in 1 episode (0.9%), while incomplete paralytic ileus in 2 (1.8%), diarrhea in 3 (2.7%), and liver dysfunction in 7 (6.4%). The entire rates turned to be 11.8% (itraconazole) versus 4.5% (posaconazole) (*P* = .049).

Before matching, 1 case (0.9%) died of drug-resistant bacterial pneumonia in the posaconazole group, while 1 case did not survive from severe pneumonia (unclear pathogen) and another failed due to severe pneumonia (unclear pathogen) and multiorgan failure in the itraconazole group (1.8%) (*P* = 1.000). None of those cases were in the matched cohorts.

## 4. DISCUSSION

This retrospective study first compared the efficacy and safety of itraconazole prophylaxis in an intravenous-oral regimen with oral suspension of posaconazole prophylaxis among patients with AL. The results indicated that either intravenous-oral itraconazole or posaconazole suspension was effective in preventing IFDs, while posaconazole suspension seemed more tolerable. Additionally, we roughly evaluated the benefit–risk ratio of these two antifungal prophylaxis strategies and found that posaconazole was superior to itraconazole in terms of benefit–risk ratio.

When it comes to the PAP, triazoles are always popular.^7,15^ In a previous study, the proportions of different PAP drugs were 75.8% (triazoles), 13.1% (echinocandins), and 11.1% (AmB).^7^ Due to the epidemiological shift toward mold infections worldwide, fluconazole has not been the preferred choice to prevent IFDs.^14^ Mold-active prophylaxis, including posaconazole, voriconazole, itraconazole, and others, has been listed in the recommendations^9,10^ and has significantly reduced the incidence of IFDs.^6,7,16^ Li et al^7^ reported that PAP decreased the rate of IFDs (6.8% vs 17.9%, *P* = .000) and the IFD-related mortality (10.1% vs 29.7%, *P* = .000). However, the choice of PAP drugs has been a popular topic for years, because of the variable efficacy among different patient groups, regions, and centers.

Posaconazole is the preferred agent due to its broad anti-mold activity and well-tolerated potentiality.^8,9,14^ In a randomized and multicenter study, proven or probable IFDs occurred in 5% (14/304) of patients in the posaconazole group, as compared with 11% (33/298) in the fluconazole or itraconazole group (*P* = .003).^12^ In another multicenter prospective study from Italy, the percentages of proven and probable IFDs were significantly different between the posaconazole prophylaxis group and the itraconazole group (18.9% vs 38.7%, *P* < .001).^17^ Besides, posaconazole prophylaxis resulted in lower mortality from any cause and longer survival free from proven or probable IFDs.^12,17^ It should also be noted that, the efficacy of itraconazole prophylaxis seemed not inferior too much.^4,17,18^ Tormo et al^18^ reported that the rates of proven and probable IFDs were 5.3% in itraconazole group versus 1.7% in posaconazole group (*P* = .095), while the possible, probable or proven IFDs were more frequent in the itraconazole group (25% vs 10%; *P* = .002). Our results, in line with this real-world observational-retrospective study, indicated an efficacy advantage of posaconazole prophylaxis only when possible, probable and proven IFDs were taken into accounts. What’s more, the overall clinical failure was lower in the posaconazole group, consistently with the previous studies.^12,18^ The percentages of overall clinical failure however were lower in our cohorts than that of previous reported, probably because the High Efficiency Particulate Air-filtered-filtered rooms prevented exposure, and the supportive therapy covered up some manifestations of adverse events. Importantly, the tolerability of posaconazole seemed better in our results, which is not in parallel with the other studies.^6,12,18^ Besides, although itraconazole has been related with more adverse events or drug–drug interactions,^19^ the rate of adverse events possibly or probably related to itraconazole seemed lower in this study than the other studies,^12,18,19^ perhaps owing to our adjustment on the dose of Vinca alkaloids and efficiently and effectively supportive therapy.

This study has some limitations. One is its retrospective nature, which may have been accompanied by selection bias and recalling bias. Therefore, it is possible that the incidence of proven, probable, or possible IFDs and clinical failure were underestimated. Another limitation is that this is a single-center study with limited cohorts. Although the PSM has been introduced to reduce the influence of bias and confounding factors, the results should be interpreted modestly. Besides, it would be more persuasive if the therapeutic drug monitoring had been done during the posaconazole or itraconazole prophylaxis.

In conclusion, this real-world analysis contributes to provide evidence for physicians in their clinical decisions. As recommended for PAP, posaconazole suspension showed good reliability, and itraconazole in our improved regimen was not worse too much either in the efficacy or the tolerability.

## ACKNOWLEDGMENTS

This work is supported by Tianjin Municipal Science and Technology Commission Grant (21JCZDJC01170); Chinese Academy of Medical Sciences Innovation Fund for Medical Sciences (2021-I2M-C&T-B-080, 2021-I2M-1-017); Haihe Laboratory of Cell Ecosystem Innovation Fund (22HHXBSS00036).

## Supplementary Material


