# Supplementary material for: Intravenous-oral itraconazole versus oral posaconazole in preventing invasive fungal diseases for acute leukemia patients
Source: Blood Sci. 2023 Mar 16;5(2):106–10. doi: 10.1097/BS9.0000000000000155 (PMC10205377; doi:10.1097/BS9.0000000000000155)
Supplement: Supplementary file 1 [file bs9-5-106-s001.pdf]

Supplementary Materials

Table 1. Details of patients with proven, probable and possible IFD.

ID Number	Age (yr)	Gender	Primary Diagnosis	Chemotherapy Phases	Duration of Neutropenia (ANC $<0.5\times10^9$/L) (days)	Severe Neutropenia (ANC $<0.1\times10^9$/L)	Prophylactic Drugs	Evidences of IFD	IFD diagnosis	Drug-sensitivity	Antifungal-Drugs Changes	Clinical Outcomes
1	M	37	AML	Induction	24	Yes	Itraconazole	Blood Culture: Candida tropicalis.	Proven IFD	Resisitant to Itraconazole.	adjusted to Amphotericin B.	IFD remitted.
2	M	45	AML	Induction	16	Yes	Itraconazole	Blood Culture: Candida tropicalis.	Proven IFD	Intermediate to Itraconazole.	adjusted to Amphotericin B and Caspofungin..	IFD remitted.

3	F	52	AML	Consolidation	13	Yes	Posaconazole	CT: segmental consolidation in the right upper lobe.	Possible IFD	NA	adjusted to Amphotericin B and voriconazole.	IFD remitted.
4	F	53	ALL	Consolidation	8	Yes	Posaconazole	CT: multiple segmental consolidation among the whole lung.	Possible IFD	NA	adjusted to voriconazole.	IFD remitted.
5	F	45	ALL	Consolidation	22	Yes	Posaconazole	CT: dense, well-circumscribed lesions without a halo sign in the left upper lobe.	Possible IFD	NA	adjusted to voriconazole.	IFD remitted.

6	M	45	AML	Induction	21	Yes	Itraconazole	CT: multiple segmental consolidation among the whole lung.	Possible IFD	NA	adjusted to voriconazole.	IFD remitted.
7	F	27	AML	Induction	25	Yes	Itraconazole	CT: dense, well-circumscribed lesions without a halo sign in the left lower lobe.	Possible IFD	NA	adjusted to Amphotericin B (intolerable) and voriconazole.	IFD remitted.
8	M	53	AML	Induction	23	Yes	Itraconazole	CT: segmental consolidations among the whole lung.	Possible IFD	NA	adjusted to voriconazole.	Died of pneumonia (unclearpathogen).

9	M	32	AML	Induction	19	Yes	Itraconazole	CT: multiple nodules and segmental consolidations in the right lung.	Possible IFD	NA	adjusted to voriconazole.	Lost to follow-up.
10	F	40	AML	Induction	24	Yes	Itraconazole	CT: segmental consolidations in the left lower lobe.	Possible IFD	NA	adjusted to voriconazole.	IFD remitted.
11	M	47	ALL	Induction	12	Yes	Itraconazole	CT: segmental consolidations and dense, well-circumscribed lesions without a halo	Possible IFD	NA	adjusted to voriconazole.	IFD remitted.

								sign among the whole lung.				
12	M	28	AML	Induction	32	Yes	Itraconazole	CT: multiple segmental consolidations among the whole lung.	Possible IFD	NA	adjusted to voriconazole.	IFD remitted.
13	F	24	AML	Induction	57	Yes	Itraconazole	CT：dense, well-circumscribed lesions in the left lower lobe.	Possible IFD	NA	adjusted to posaconazole (not improved on CT) and then voriconazole.	IFD remitted.

14	F	28	ALL	Induction	38	Yes	Itraconazole	CT: segmental consolidation in the left upper lung.	Possible IFD	NA	adjusted to voriconazole.	IFD remitted.
15	F	13	AML	Induction	28	Yes	Itraconazole	CT: multiple segmental consolidations in the right lung.	Possible IFD	NA	adjusted to voriconazole	IFD remitted
16	F	55	AML	Induction	12	Yes	Itraconazole	CT：dense, well-circumscribed lesion without a halo sign and then consolidated.	Possible IFD	NA	adjusted to voriconazole	IFD remitted

17	F	38	AML	Induction	8	Yes	Itraconazole	CT: segmental consolidation in the right upper lobe.	Possible IFD	NA	adjusted to voriconazole	IFD remitted
18	F	47	AML	Consolidation	30	Yes	Itraconazole	CT: dense, well-circumscribed lesions without halo signs and partly consolidated.	Possible IFD	NA	adjusted to voriconazole (not improved on CT) and then Amphotericin B	IFD remitted
19	M	19	ALL	Consolidation	6	Yes	Itraconazole	CT: segmental consolidations in the lower lobes.	Possible IFD	NA	adjusted to voriconazole.	IFD remitted

20	M	15	AML	Consolidation	13	Yes	Itraconazole	CT: segmental consolidations and cavities in the left upper lobe.	Possible IFD	NA	adjusted to voriconazole	IFD remitted
21	M	30	AML	Consolidation	24	Yes	Itraconazole	CT: segmental consolidations in the upper lobes.	Possible IFD	NA	adjusted to voriconazole	IFD remitted

Abbreviations: *AML = acute myeloid leukemia, ALL = acute lymphocytic leukemia, ANC = absolute neutrophil count, CT= computed tomography, F=female, ID=identity, IFD=invasive fungal disease, M=male, NA=not available, yr=years old.*
